# PET/CT imaging for evaluation of multimodal treatment efficacy and toxicity in advanced NSCLC—current state and future directions

**DOI:** 10.1007/s00259-021-05211-8

**Published:** 2021-03-24

**Authors:** Chukwuka Eze, Nina-Sophie Schmidt-Hegemann, Lino Morris Sawicki, Julian Kirchner, Olarn Roengvoraphoj, Lukas Käsmann, Lena M. Mittlmeier, Wolfgang G. Kunz, Amanda Tufman, Julien Dinkel, Jens Ricke, Claus Belka, Farkhad Manapov, Marcus Unterrainer

**Affiliations:** 1 Department of Radiation Oncology, University Hospital, LMU Munich, Munich, Germany; 2 Medical Faculty, Department of Diagnostic and Interventional Radiology, University Dusseldorf, D-40225 Dusseldorf, Germany; 3 German Cancer Consortium (DKTK), partner site Munich; and German Cancer Research Center (DKFZ), Heidelberg, Germany; 4 Comprehensive Pneumology Center Munich (CPC-M), Member of the German Center for Lung Research (DZL), Munich, Germany; 5 Department of Nuclear Medicine, University Hospital, LMU Munich, Munich, Germany; 6 Department of Radiology, University Hospital, LMU Munich, Munich, Germany; 7 Division of Respiratory Medicine and Thoracic Oncology, Department of Internal Medicine V, Thoracic Oncology Center Munich, University of Munich (LMU), Munich, Germany; 8 Department of Radiology, Asklepios Lung Center Munich-Gauting, Munich, Germany

**Keywords:** PET, Lung cancer, Radiation oncology, Immunotherapy

## Abstract

**Purpose:**

The advent of immune checkpoint inhibitors (ICIs) has revolutionized the treatment of advanced NSCLC, leading to a string of approvals in recent years. Herein, a narrative review on the role of 18F-fluorodeoxyglucose positron emission tomography/computed tomography (FDG PET/CT) in the ever-evolving treatment landscape of advanced NSCLC is presented.

**Methods:**

This comprehensive review will begin with an introduction into current treatment paradigms incorporating ICIs; the evolution of CT-based criteria; moving onto novel phenomena observed with ICIs and the current state of hybrid imaging for diagnosis, treatment planning, evaluation of treatment efficacy and toxicity in advanced NSCLC, also taking into consideration its limitations and future directions.

**Conclusions:**

The advent of ICIs marks the dawn of a new era bringing forth new challenges particularly vis-à-vis treatment response assessment and observation of novel phenomena accompanied by novel systemic side effects. While FDG PET/CT is widely adopted for tumor volume delineation in locally advanced disease, response assessment to immunotherapy based on current criteria is of high clinical value but has its inherent limitations. In recent years, modifications of established (PET)/CT criteria have been proposed to provide more refined approaches towards response evaluation. Not only a comprehensive inclusion of PET-based response criteria in prospective randomized controlled trials, but also a general harmonization within the variety of PET-based response criteria is pertinent to strengthen clinical implementation and widespread use of hybrid imaging for response assessment in NSCLC.

## Introduction

Intrinsic genomic instability in non-small cell lung cancer (NSCLC) has facilitated resistance to cytotoxic or targeted therapies. The advent of immune checkpoint inhibitors (ICIs) has revolutionized the treatment of advanced/metastatic NSCLC.

Immune checkpoints are inhibitory pathways that are pertinent to self-tolerance. Tumors confer immune resistance by interference with these pathways. As a means of counterpoise, ICIs which act by inhibiting these specific inhibitory immune checkpoints were developed. Agents targeting cytotoxic T lymphocyte-associated antigen 4 (CTLA-4) [ipilimumab] and programmed cell death receptor 1 (PD-1) (nivolumab, pembrolizumab) or its ligand programmed cell death ligand 1 (PD-L1) [durvalumab, atezolizumab] have been approved for treatment of advanced NSCLC [1]. This marks the dawn of a new era bringing forth new challenges vis-à-vis treatment response assessment with observation of novel phenomena due to their mechanism of action. These patterns of response include durable response, hyperprogression, pseudo-progression, and dissociated response possibly amenable to local ablative therapies [2]. Moreover, new patterns of systemic side effects, i.e., immune-related adverse events (irAEs), are accompanying these therapeutic effects. However, only a small proportion of patients actually respond to treatment with ICIs [3]. The assessment of PD-L1 expression—despite its inherent limitations—is widely regarded as the best available predictive biological biomarker and the search for more robust biomarkers remains an area of intensive research [4]. Over the years, there has been increased interest in molecular imaging, particularly ^18^F-fluorodeoxyglucose positron emission tomography/computed tomography (FDG PET/CT) as a tool for response assessment and prognostication beyond the mere morphological assessment using CT and MRI [5].

In recent years, a number of groups have proposed modification of current criteria to more robustly assess response to these novel agents. In 2019, the EANM (European Association of Nuclear Medicine) published a consensus report addressing this issue [6]. Herein, we highlight the current state, limitations, and future directions of hybrid imaging for treatment planning, evaluation of multimodal treatment efficacy and toxicity in inoperable locally advanced and metastatic NSCLC.

## Current multimodal treatment strategy

### Inoperable locally advanced (stage III) disease

Radiotherapy is a fundamental pillar of cancer therapy and has been demonstrated to induce both local and systemic immune responses. Briefly, irradiation triggers the release of damage-associated molecular patterns (DAMPs) that can promote an immunogenic response, described as in situ vaccination [7]. In inoperable stage III NSCLC, curative-intent platinum-based chemoradiotherapy (CRT) followed by consolidation ICI with the PD-L1 inhibitor durvalumab is the new standard of care (SoC) [8]. Furthermore, there are a plethora of studies currently assessing this tri-modal treatment paradigm in the concurrent or sequential setting [9].

### Metastatic (stage IV) disease

In the metastatic setting, pre-clinical studies and case reports have demonstrated a phenomenon whereby shrinkage of untreated tumors occurs concurrently with shrinkage of tumors receiving localized radiotherapy, a phenomenon described as the “abscopal effect” [10–13]. In addition, in the oligo-metastatic setting, patients on systemic treatment (including ICIs) might be amenable to local ablative/consolidative therapies [14, 15].

Nivolumab was the first ICI approved by the US Food and Drug Administration (FDA) in 2015 for advanced or metastatic (m)NSCLC in the second-line setting. Later that year, pembrolizumab was granted accelerated approval in the second line. Atezolizumab was also added to the repertoire in the second-line setting for PD-L1 unselected patients with or without epidermal growth factor receptor (EGFR) or anaplastic lymphoma kinase (ALK) alterations the following year, and shortly after, pembrolizumab was the first drug approved in the first-line treatment for non-oncogene addicted patients with PD-L1 tumor proportion score (TPS) ≥ 50% and expansion of this indication in 2019 to include patients with PD-L1 positive tumors based on the KEYNOTE-042 trial [1].

PD-L1 expression can either be constitutive or induced in many tumors to promote cancer immune evasion. In an attempt to combat this adaptive immune resistance, combinations with chemotherapy and anti-angiogenic agents have also received FDA approval [1]. The latest addition to this set of therapeutics is nivolumab plus ipilimumab (approved on May 15, 2020 for first-line treatment of non-oncogene addicted PD-L1 positive metastatic/recurrent NSCLC), atezolizumab monotherapy (approved on May 18, 2020 in the first-line for mNSCLC with PD-L1 ≥ 50% of tumor cells or PD-L1 tumor-infiltrating immune cells covering ≥ 10% of the tumor area and no EGFR or ALK genomic tumor aberrations), and nivolumab/ipilimumab plus 2 cycles of platinum-doublet chemotherapy (approved on May 26, 2020 for the first-line treatment of non-oncogene addicted metastatic/recurrent NSCLC irrespective of histology and PD-L1 expression based on the results of CheckMate 9LA) [16–18].

## Imaging in NSCLC

### Standard conventional imaging

Historically, in an attempt to standardize response assessment in oncological patients, the World Health Organization (WHO) initially published recommendations in the 1979 WHO Handbook and sanctioned a publication by Miller et al. in 1981, ultimately evaluating response assessment based on bi-dimensional tumor measurements [19]. However, the WHO criteria had some major caveats in that the number of measurable lesions and minimum measurable size of lesions were not adequately defined.

Two decades later, joint CT-based criteria by the “European Organization for Research and Treatment of Cancer” (EORTC) and National Cancer Institute (NCI) were published—Response Evaluation Criteria in Solid Tumors” (RECIST). The RECIST criteria proposed a uni-dimensional (measurement of longest tumor diameter) model integrating a combined assessment of all existing lesions, characterized by target lesions and non-target lesions [20]. A revised version RECIST 1.1 ensued in 2009 and currently represents the gold standard in CT-based assessment with the majority of (C)RT/ICI clinical trials evaluating response based on these criteria [21].

Clinical response patterns to immunotherapy are more complex than those to cytotoxic or targeted agents. Hence in 2009, immune-related response criteria (irRC) were the premier novel immune therapy criteria proposed to cover additional patterns of response observed with these agents and was based on bi-dimensional measurements [22]. Refinement led to additional immune-related criteria, namely, immune-related RECIST (irRECIST), effectively adapted from RECIST and based on uni-dimensional measurements, proven superior to irRC [23, 24]. Thereafter, in an attempt to promote congruence, the RECIST working group proposed a consensus guideline—immune RECIST (iRECIST) based on RECIST 1.1 [25], and finally in 2018, the immune-modified RECIST (imRECIST) criteria designed to better encapsulate response to immunotherapy were proposed [26]. A summary of available CT-based criteria is presented in Table 1, see also analogously (Citation Reference [27]).

**Table 1:** Overview CT-based criteria for response assessment

	RECIST 1.1 (2009)	irRC (2009)	irRECIST (2013)	iRECIST (2017)	imRECIST (2018)
Complete response	• Disappearance of all TL/NTL • Nodal SAD < 1.0 cm • No new lesion	• Disappearance of all lesions (measurable or not) • No new lesions • Confirmation by consecutive CSI control in ≥ 4 weeks	• Disappearance of all TL/NTL • Nodal SAD < 1.0 cm • No new lesion	• Disappearance of all TL/NTL • Nodal SAD < 1.0 cm • No new lesion	• Disappearance of all TL/NTL • Nodal SAD < 1.0 cm • No new lesion
Partial response	• ≥ 30% decrease relative to baseline • No new lesion	• ≥ 50% relative to baseline • Confirmation by consecutive CSI control in ≥ 4 weeks	• ≥ 30% decrease relative to baseline • No new lesion	• > 30% decrease relative to baseline • No new lesion	• ≥ 30% decrease relative to baseline • No new lesion
Stable Disease	• Neither CR, PR nor PD	• Neither CR, PR nor PD	• Neither CR, PR nor PD	• Neither CR, PR nor PD	• Neither CR, PR nor PD
Progressive Disease	• ≥ 20% increase relative to baseline • Or progression of NTL • Or new lesion	• ≥ 25% increase relative to nadir • New lesions added to tumor burden • Confirmation by consecutive CSI control in ≥4 weeks	• ≥ 20% increase • And ≥ 5 mm absolute increase in total measured tumor burden relative to nadir(i. *E. minimum* recorded tumor burden). • Confirmation of progression in ≥4 weeks after suspected PD	iUPD: PD RECIST 1.1 iCPD: • Confirmation 4–8 weeks later • Any further size increase in TL sum >5 mm • Any progression of NTL • Any further size increase of the sum of new TL > 5 mm • Appearance of another new lesion	• ≥ 20% increase of summed tumor burden of TL including new lesions compared to nadir • New lesions/NTL progression do not necessarily define PD • Negated in a subsequent follow-up ≥ 4 weeks with non-PD
Reference	[21]	[22]	[23, 24]	[25]	[26]

Abbreviations: *TL* target lesion; *NTL* non-target lesion; *SAD* short-axis-diameter, *CSI* cross sectional imaging; *PD* progressive disease; *iUPD* unconfirmed PD; *iCPD* confirmed PD

### Novel phenomena of response to ICIs

Owing to their mechanism of action, ICIs have demonstrated novel patterns of response [2]. Briefly, the following phenomena have been observed.

#### Durable response

In the first instance, in heavily pretreated patients with advanced NSCLC receiving nivolumab for up to 96 weeks, the estimated 5-year OS rate was 16%, despite nivolumab discontinuation after a maximum of 96 weeks [28]. This is an interesting observation and poses the relevant question of frequency and duration of ICI treatment also from a cost-effectiveness and health economics standpoint and remains a topic of intense investigation.

#### Pseudo-progression

A phenomenon is characterized by a temporary increase in tumor burden, possibly due to transient immune-cell infiltrate, therapy-related necrosis, and edema followed by tumor regression. In NSCLC, rates of up to 7% have previously been described. This poses a major challenge, since the risk of misinterpreting treatment response based only on size (RECIST/WHO criteria) is high [19, 21, 29]. The additional application of FDG PET providing functional information would suggest improved differentiation between pseudo- and true progression. However, due to the complexity of the tumor microenvironment and involved stromal cells, enhanced FDG uptake could still mimic an aggregation of proliferating tumor cells similar to an elevated uptake seen post-(chemo-)radiotherapy and representing an influx of inflammatory cells. Indeed modified PET criteria were recently published and demonstrated a decent ability in predicting clinical outcome and are described in more detail below [30]. Nevertheless, due to rarity of this phenomenon, while discontinuing treatment might be detrimental, more often than not true progression is the case.

#### Hyperprogression

In contrast, hyperprogression is characterized by acceleration of disease. In a French multicenter retrospective study, hyperprogressive disease was observed in pretreated patients with advanced NSCLC who received PD-1/PD-L1 inhibitors or single agent chemotherapy in 13.8% and 5.1%, respectively [31]. More recently, different definitions for hyperprogressive disease were assessed in a pooled retrospective trial with the authors determining that the 5 definitions assessed did not characterize the same tumoral behavior with incidences ranging from 5.4–18.5%. A novel definition characterized by the difference between tumor growth rate pre- and during therapy > 100 was proposed [32]. Further validation is required and utility of FDG PET in this scenario warrants further investigation.

#### Dissociated response

Another observed phenomenon is the concurrent growth and regression of different lesions corresponding to mixed response seen under chemotherapy or targeted therapies. In a monocentric study of advanced NSCLC patients treated with PD-1/PD-L1 inhibitors, dissociated response occurred in 8% of the cohort and was associated with improved survival in comparison to patients with true progression [33]. These progressive lesions are potentially amenable to local ablative therapies with potential induction of “the abscopal effect” in patients treated with radiotherapy.

### PET imaging in advanced NSCLC

#### PET in NSCLC diagnosis

FDG PET/CT is recommended as the first-line staging modality especially in potentially curable NSCLC due to its excellent diagnostic accuracy [34]. It combines the strength of FDG-PET to visualize cells with an elevated glycolytic rate, which is an important hallmark of cancer cells, and the high spatial resolution of the CT. The high negative predictive value for detection of (thoracic lymph node metastases) has a major impact on initial patient management [35].

The field of radiogenomics is an emerging area of interest. Several studies have demonstrated a favorable performance of PET/CT radiomic features in predicting mutational status [36–38].

#### PET for treatment planning in curative-intent locally advanced (LA)-NSCLC

The largest prospective multicenter study to date was recently published assessing the role of FDG PET/CT for tumor volume delineation (TVD) in patients with inoperable LA-NSCLC undergoing CRT. In the study, PET-alone TVD potentially improved local control without compromising toxicity thus corroborating the lack of additional benefit with elective nodal irradiation observed in previous smaller trials [39].

Emphasizing the role of PET-based dose escalation, toxicity data of a randomized phase II European study, the PET-boost trial was published last year [40]. Patients with stage II/III NSCLC were randomized and treated with an isotoxic simultaneous integrated boost (SIB) ≥ 72 in 24 daily fractions Gy to the planning target volume (PTV) of the entire primary tumor (arm A: 54 patients) vs. only to the regions within the PTV with an SUVmax ≥ 50% on pretreatment FDG PET/CT (arm B: 53 patients). Due to slow accrual, the trial was terminated early after randomization of 107 patients (target: 164). Seventeen deaths with 13 possibly treatment related occurred in the cohort of 107 patients [40]. In addition, results of the primary endpoint of 1-year freedom from local failure (FFLF) and secondary endpoint of overall survival were presented at the European Society for Radiotherapy and Oncology virtual meeting 2020 (28 November 2020 - 01 December 2020): at a median follow-up of 12.6 months, 1-yr FFLF rates were 97% in arm A and 91% in arm B; 1-/3-year overall survival (OS) rates were 77%/37% in arm A and 62%/33% in arm B [41]. In another non-randomized phase II study adopting a different strategy, mid-treatment PET-based TVD was feasible and associated with favorable loco-regional tumor control in patients receiving (C)RT for inoperable stage II/III disease [42] and is currently being assessed in a randomized manner in the Radiation Therapy Oncology Group (RTOG) 1106/ACRIN 6697 trial (NCT01507428). Also, the results of this trial were recently presented at the 2020 virtual World Conference on Lung Cancer (28-31 January 2021): a total of 138 stage III patients were randomized 1:2 to a standard 60-Gy arm vs. an adaptive arm delivered in 30 daily fractions. The primary endpoint was 2-year local-regional control rate. Median prescription dose was 71 Gy in the adaptive arm. No significant difference in grade ≥ 3 radiotherapy-induced toxicity was noted. The overall 2-year local-regional tumor progression-free time was 27.5 vs. 28.4 months in the standard and adaptive arm, respectively. This study demonstrated safety and feasibility of PET-based dose escalation in stage III disease (published abstract not yet available). 

#### PET for treatment planning in metastatic NSCLC

Stereotactic ablative radiotherapy (SABR) in combination with a highly tumor-selective immunocytokine—a form of interleukin 2 (IL2), namely, L19-IL2: a substance consisting of the single-chain (scFv) tumor-specific human antibody L19 targeting extra-domain B (ED-B) explicitly (anti-ED-B scFv L19) coupled to IL2 demonstrated some promising results in a phase I trial in the absence of any severe (grade ≥ 3) toxicity (NCT02086721). The results have been proven sufficiently robust to support progression to a randomized phase II study—ImmunoSABR: an open-label, multicenter, randomized controlled phase II trial assessing SoC treatment (including ICI) vs. SoC plus SABR/L19-IL2 in limited metastatic NSCLC. Importantly all participants will undergo FDG PET/CT before randomization and contrast-enhanced CT-scans on follow-up. Hence, PET/CT with its superior diagnostic accuracy will more accurately filter out patients that do not meet the inclusion criteria (stage IV disease and a max. of 10 metastases) and enable better stratification of oligo- (max. 5 metastases) vs. poly-metastatic (6–10 metastases) disease.

Response assessment will be performed per blinded radiological review for every scan and assessed per RECIST 1.1 and exploratory iRECIST. The primary endpoint of the study is progression-free survival (PFS) at 1.5 years and exploratory endpoints will include radiomics analysis (hypoxia status, response prediction) and response assessment per iRECIST. This will certainly provide valuable insights in validation of this criterion [43].

#### PET for prediction of outcome in advanced NSCLC

Pre-treatment FDG-PET parameters have been demonstrated as reliable prognostic factors for outcome and survival. While SUVmax is mostly used for assessment of treatment response, metabolic tumor volume (MTV), total MTV (TMTV), and total lesion glycolysis (TLG) are considered to be the strongest prognosticators at initial staging. Several studies have shown the association of these parameters with outcome [44, 45]. An association of TMTV and inflammatory status with poor outcome and lack of durable clinical benefit (DCB) has been described [44]. In another study, radiomic features from baseline pre-treatment FDG PET/CT could reliably identify patients most likely to achieve a DCB [46]. However, it has to be stated that there is no widespread and uniform application of these parameters in clinical routine.

#### PET for response assessment to (C)RT

**Fig. 1:**
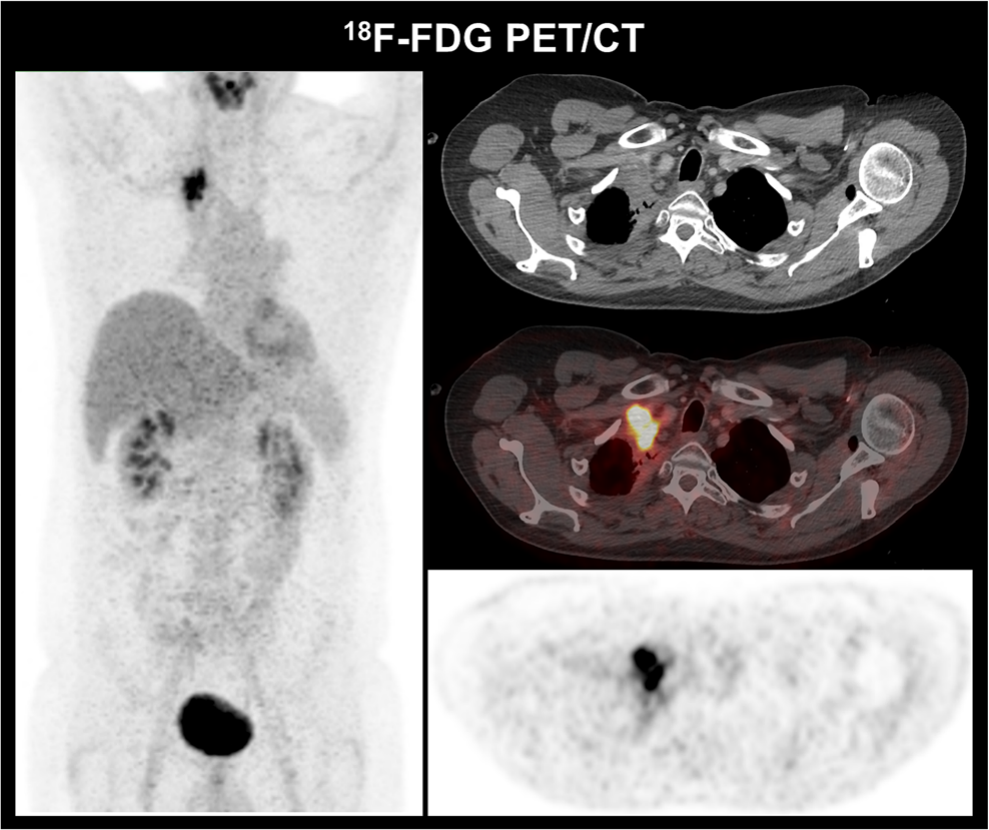
Treatment response assessment in a 51-year-old patient with NSCLC cT3 N2 M0 (TNM 8th edition). Following definitive chemoradiation to a total dose of 63.6 Gy and concomitant cisplatin/vinorelbine, consolidation with durvalumab was initiated. Previous CT-staging scans were suggestive of post-treatment and treatment-related changes in the right upper lobe; 17 months after therapy initiation, FDG PET/CT, however, revealed a tumor recurrence with highly elevated tumor metabolism which was later confirmed by histopathology

The changes in metabolic activity following (C)RT can be observed earlier than morphologic changes on CT-scans and metabolic changes characterized by PET-metrics (SUVmax; MTV; TLG) during or shortly after treatment have been identified as prognostic biomarkers for disease recurrence and survival [47–55]. An association of residual MTV at a cutoff of 25cm^3^ with tumor local control was identified [52] and corroboration of these findings in addition to the prognostic role of pre-treatment primary tumor (PT)-MTV, reduction in mid- to post-PT-MTV, and an association between post-treatment PT-MTV and outcome has been previously published [49, 50]. Furthermore, with regard to the role of primary tumor vs. lymph node metastases metrics, van Diessen et al. detected an association and superiority of post-treatment primary tumor PET-metrics compared to lymph node metrics in predicting outcome [54]. An exemplary patient can be seen in Fig. 1.

#### PET for response assessment to immunotherapy

Back in 1999, there were first attempts to standardize PET-based response assessment in oncological imaging; the EORTC firstly used standardized metabolic information in specified criteria for the response assessment of oncological diseases in general [56]. Of note, the EORTC criteria were also the first PET-based criteria to be applied for monitoring of immunotherapy [57]. These criteria were then refined with proposal of the “PET Response Criteria in Solid Tumors” (PERCIST 1.0) criteria published by Wahl et al. in 2009 [58]. The major novelty of these criteria was the introduction of SUL (i.e., standardized uptake value [SUV] corrected for the lean body mass) as an imaging parameter. Here, tumor SUL 1.5-fold higher than the SUL of the non-affected liver was set as a prerequisite for an evaluable lesion and assessed within a spherical volume of interest in the lesion with the most intense uptake.

Along with the rise of immunotherapeutic agents, these PET-based criteria however had to be refined as well, in order to adapt to the new clinical phenomena that accompanied immunotherapeutic agents. Therefore, Cho et al. prospectively compared different response criteria (i.e., RECIST 1.1, irRC, EORTC, and PERCIST 1.0) in a rather small set of patients undergoing immunotherapy in order to derive and evaluate an optimized set of parameters comprising morphological and metabolic response parameters on PET/CT during immunotherapy. The best combination of both morphological and functional parameters was subsequently summated into new criteria “PET/CT Criteria for Early Prediction of Response to Immune Checkpoint Inhibitor Therapy” (PECRIT) [59].

Additional criteria for response assessment to immunotherapy were suggested by the Heidelberg group. After evaluating the pattern of response in melanoma patients, the “PET Response Evaluation Criteria for Immunotherapy” (PERCIMT) were established, which take into account the clinical relevance of the absolute number of new lesions during immunotherapy as a definite prerequisite for defining progression rather than a mere increase of PET-based parameters [60, 61].

Most recently, immunotherapy adopted PERCIST criteria, i.e., iPERCIST, were proposed. Of note, these criteria were derived from a set of patients with advanced NSCLC undergoing nivolumab therapy. Here, a modification of the PERCIST criteria with features of iRECIST criteria demonstrated a good stratification of patients with improved clinical outcome; vice versa, therapy failure was also better captured [30]. In sum, the term unconfirmed progressive metabolic disease (UPMD) was introduced; in case of UPMD, an additional PET/CT scan is needed leading either to the classification of confirmed progressive metabolic disease (CPMD) or to omission of UPMD in case of subsequent metabolic response to immunotherapy [30].

To summarize, several novel criteria for response assessment based on PET-imaging have been proposed and further refined with special emphasis on clinical phenomena that accompany immunotherapy. However, these criteria have to be incorporated into randomized, clinical trials to confirm their final validity in prospective settings and comprehensively foster wide-spread use of hybrid imaging (see Table 2), see also analogously (Citation Reference [27]).

**Table 2:** Overview PET(/CT)-based criteria for response assessment

	EORTC (1999)	PERCIST (2009)	PECRIT (2017)	PERCIMT (2018)	iPERCIST (2019)
Modality	PET	PET	PET/CT	PET/CT	PET/CT
Complete response	• Reduction of FDG uptake to background levels	• Reduction of FDG uptake to the level of background blood pool	• Disappearance of all metabolically active tumors and TL • SAD reduction target lymph nodes < 10 mm • No new lesions	• Complete resolution of all FDG-avid lesions • No new FDG-positive lesion	• Reduction of FDG uptake to the level of background blood pool
Partial response	• ≥ 15% reduction of FDG uptake	• ≥ 30% reduction in SUL_peak_ • Min. 0.8 units of measurable lesions	• ≥ 30% reduction in SUL_peak_ • ≥ 30% decrease in TL diameter sum	• Complete resolution of some FDG-avid lesions • No new FDG-positive lesion	• ≥30% reduction in SUL_peak_
Stable disease	• Neither CR, PR nor PD	• Neither CR, PR nor PD	• Neither CR, PR nor PD	• Neither CR, PR nor PD	• Neither CR, PR nor PD
Progressive disease	• ≥ 25% increase of FDG uptake	• > 30% increase in SUL_peak_ • Min. 0.8 units of measurable lesions	• > 30% increase in SUL_peak_ • Or new metabolically active lesion • ≥ 20% increase in target lesion diameter (Min. 5 mm) • Or new lesions	• Four or more new lesions of < 10 mm functional diameter • Or three or more new lesions >10 mm functional diameter • Or two or more new lesions > 15 mm functional diameter	UMPD: • ≥ 30% increase in SUL_peak_ • or new FDG-positive lesion CPMD: • Confirmation of progression by second PET after 4–8 weeks later • Otherwise reset in case of PMR or SMD
Reference	[56]	[58]	[59]	[60, 61]	[30]

Abbreviations: *TL* target lesion; *SUL* SUV corrected for lean body mass; *SAD* short-axis-diameter, *UPMD* unconfirmed progressive metabolic disease; *CPMD* confirmed progressive metabolic disease; *PMR* partial metabolic response; *SMD* stable metabolic disease

Taking a closer look at current clinical trials, in a study including 72 patients with advanced pretreated NSCLC on nivolumab, an additional prognostic value of metabolic response assessment was postulated, potentially aiding treatment decision-making [62]. In another study, changes of FDG-uptake in terms of PERCIST criteria (compared to the morphological changes on RECIST 1.1 criteria) were predictive of treatment efficacy even at an early stage of 1 month after initiation of nivolumab in NSCLC patients; this feature was also shown to be an independent prognostic factor in multivariate analysis [63]. Also, response on FDG PET (using EORTC criteria) in NSCLC patients undergoing atezolizumab therapy 6 weeks after initiation was predictive of further disease course [64]. Additionally, FDG PET imaging as follow-up in patients classified as progressive disease (PD) per PERCIST criteria identified patients with pseudo-progression and immune dissociated-response in more than half of patients previously classified as PD; a significantly improved clinical outcome was observed in these patients [65].

**Fig. 2:**
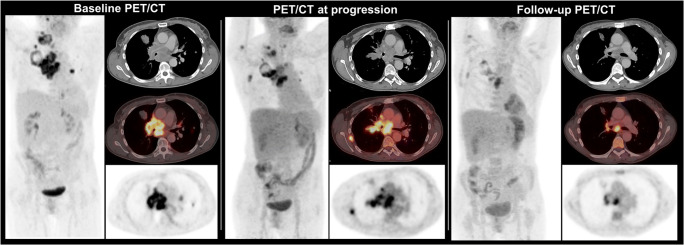
Treatment response assessment in a 49-year-old patient with NSCLC cT2b N3 M1 (TNM 8th edition) with extensive progressive disease (rib metastasis) 2 months after initiation of combined systemic treatment with carboplatin/pemetrexed/pembrolizumab. Consecutively, treatment was modified to carboplatin/paclitaxel/atezolizumab/bevacizumab; 8 weeks after initiation, a partial response was visible on FDG PET/CT with significantly decreasing metabolic tumor volume

Interestingly, a recent abstract presented at the 2020 American Society of Clinical Oncology meeting assessed the role of residual metabolic volume in patients receiving CRT vs. CRT + ICI and only found a prognostic role of residual PT-MTV in the CRT cohort [66]. An exemplary case is displayed in Fig. 2.

Hence, additional functional imaging associated with a higher resolution for potential patient stratification into different prognostic groups helps define patients who might benefit from treatment modification or intensification. Please refer for more detailed literature [27].

#### PET for imaging immunotherapy side effects in NSCLC

A wide range of immune-related adverse events have been reported and could involve nearly every organ system but particularly endocrine, cutaneous, and gastrointestinal systems [67]. Furthermore, they can occur at any time, beginning immediately after initiation of therapy until long after completion [68]. The overall incidence of irAE for patients undergoing immunotherapy is about 25%. For NSCLC patients, the most common irAEs associated with nivolumab are rash and diarrhea, and those associated with pembrolizumab are hypo- and hyperthyroidism [69]. The importance of early detection of irAEs is essential to reduce associated morbidity. Interestingly, in the phase I CA209-003 trial, in patients receiving nivolumab, overall survival was significantly prolonged among patients with irAEs of any grade [70].

Inflammatory reactions are accompanied by irAEs and consequently lead to an elevated FDG-avidity [71], which might possibly lead to a misinterpretation of the respective PET scan despite certain temporal adaptions of FDG-uptake [72, 73]. Vice versa, this partly high FDG-avidity accompanying irAEs consequently enables localization and identification [74]; this feature gains further importance keeping in mind the association (the occurrence of) irAEs and the therapeutic efficacy of immunotherapy [75, 76].

Recently, the report from the EANM symposium on immunotherapy stated that incidental findings of irAEs should be reported, although irAEs are not necessarily associated with clinical features. However, detection of irAEs might lead to clinical interventions. Newly developed signs of irAEs have to be compared to the particular baseline scan to be able to relate these findings to immunotherapy [6].

Particularly in NSCLC patients, the occurrence of immune-related “sarcoid-like reactions” has to be kept in mind as these may be misread as progressive disease since sarcoid-like reactions consist of lymphadenopathy and pulmonary granulomatosis with high FDG-avidity [77].

**Fig. 3:**
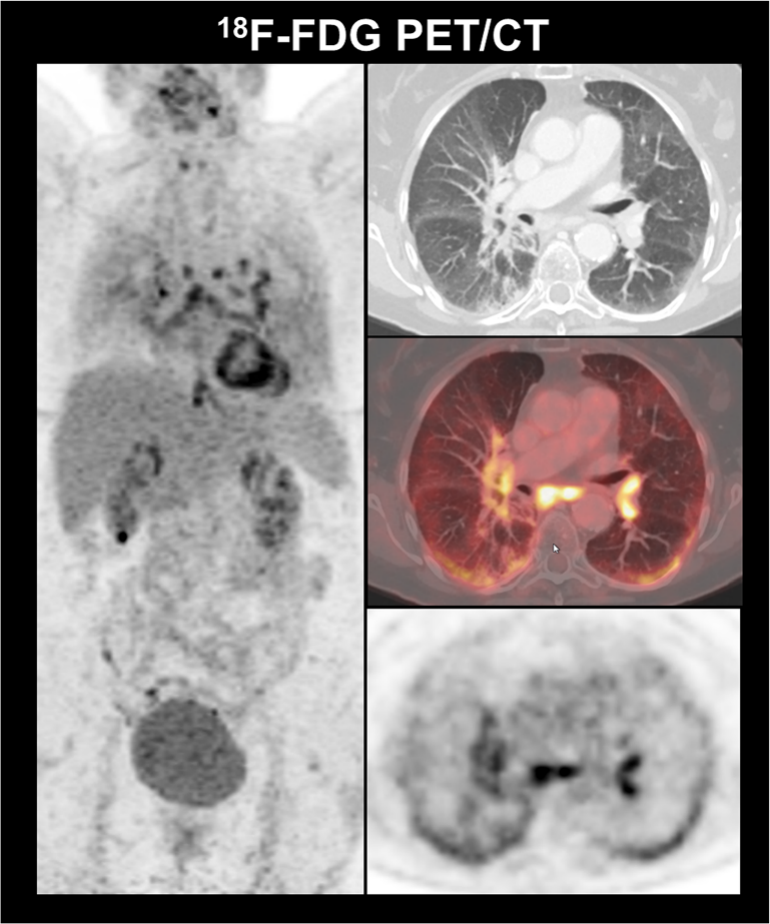
Identification of irAEs with FDG PET/CT in a 73-year-old patient with NSCLC cT3 N2 M0 (TNM 8th edition) who initially received concurrent chemoradiation with cisplatin/vinorelbine to a total dose of 63.6 Gy and was started on consolidation durvalumab. On follow-up FDG PET/CT, large ground glass opacities with consecutively elevated FDG-uptake were seen in both lungs. Moreover, newly enlarged and FDG-avid hilar lymph nodes could be observed. In sum, the findings were suggestive of immune-related pneumonitis and reactive lymphadenopathy. The patient was asymptomatic (grade 1) and as such no intervention was indicated.

The possible advantage of integration of FDG PET/CT in this scenario is the facilitation of and early detection of irAEs consequently leading to an early intervention when necessary and potential reversibility. An example of irAEs detected by FDG PET/CT is displayed in Fig. 3. Please refer for more detailed literature [27].

## Future directions

### Technological advances

Beyond the mere pharmacological and clinical advances for cancer treatment and patient care, there are, however, dedicated technological advances regarding imaging technical and data evaluation that will foster advances in research and will gain further access in clinical routine. Advanced image evaluation methods such as the extraction and analysis of radiomic features are gaining increasing importance predominantly in the research setting with the final goal to improve the clinical decision-making by improving the diagnostic accuracy of imaging beyond the mere morphological extent, but also to provide more precise predictive/prognostic information for clinical routine [78]. So far, however, despite increasing scientific recognition, there is no high level of standardization of radiomics analysis, which hampers comparability and high-throughput mining of quantitative image information from routine imaging [79]. Nonetheless, more studies correlating radiomic features with clinical outcome in NSCLC patients also undergoing combined chemoradiotherapy/immunotherapy were published recently underlining radiomics as an intensive field of research in NSCLC imaging [80, 81].

Moreover, the clinical use of artificial intelligence (AI) applications is also one of the current technological topics that increasingly arise in scientific literature. AI might be used as a tool to augment the clinical radiographic assessment of diseases such as NSCLC by applying advanced computational analyses in order to improve the accurate detection, but also the disease characterization and response assessment. In the light of evermore-increasing quantitative clinical data, but also radiographic information including advanced radiomics analyses, AI might facilitate the specific qualitative interpretation of oncologic imaging, e.g., by automated delineation, deriving outcome models or mere response assessment. Beyond the mere human assessment of radiographic data, AI and machine learning applications might potentially facilitate the interpretation of large-scale information and might positively influence clinical decision-making [82–84].

Additionally, there are specific technological PET/CT advances; whole body PET imaging represents a very promising technological advancement that might have a strong impact on hybrid imaging, as scan times can drastically be reduced compared to last-generation PET/CT scanners. Moreover, the resolution of range of whole-body PET scanners can also be used for significant reductions of the necessary doses of particular radioligands, but also for whole-body distribution and pharmacodynamic studies. These properties might improve clinical routine, but might also give new insights in research topics such as immune-directed PET. However, only a few scanners are available so far due to limited access and extensive need of resources [85–88].

### Novel ligands (e.g., PD1-targeted imaging)

Beyond the scope of glucose-based imaging, novel molecular radioligands that directly target key molecules within immune-checkpoint pathways and immune response cascades have emerged [89, 90]. So far, anti-PD-1 antibodies were predominantly labeled with ^89^Zr or ^64^Cu, an approach highly feasible for in vivo imaging PD-1–expressing tumor-infiltrating lymphocytes [90]. This represents a very promising approach for noninvasive visualization and quantification of PD-1-expression, as histochemical analyses are primarily limited by the heterogeneous tissue expression on biopsies or single tissue specimens [91]; this phenomenon is not restricted to NSCLC patients, but is the case for almost any solid tumor.

Beyond the scope of preclinical trials, several studies were already performed in humans; a study by Niemeijer et al. applied the radiolabeled anti-PD-1 monoclonal antibody ^89^Zr-nivolumab in patients with advanced NSCLC. They could demonstrate that the tumor uptake of ^89^Zr-nivolumab was significantly higher in patients with immunohistochemically proven PD-1-positive tumor-infiltrating immune cells compared to those tumors classified as PD-1-negative tumors. Of note, PD-(L)1 PET-CT could identify a highly heterogeneous tumor uptake not just on an inter-individual but also on an intra-individual basis. Particularly, highly diverging uptake between different intra-individual tumor lesions was found [92]. In further clinical studies, high uptake on pre-treatment ^89^Zr-atezolizumab PET showed a significantly stronger correlation with the individual clinical course compared to immunohistochemistry-based or RNA-sequencing-based biomarkers prior to the initiation of PD-L1-targeted therapies [93].

With special regard to immuno-PET imaging in NSCLC patients, several clinical trials are currently underway evaluating several novel ligands such as ^89^Zr-avelumab (NCT03514719, PINNACLE), ^89^Zr-durvalumab (2015-005765-23), ^99m^Tc-anti-PD-L1 (sdAb) single-photon emission computed tomography (SPECT) (NCT02978196), or combined imaging with ^89^Zr-durvalumab PET and ^111^In-CD8 T cell SPECT (NCT03853187, DONAN).

Beyond PD-(L)1 imaging with PET, several further biomarkers were deemed as potential targets for molecular imaging, predominantly in preclinical settings; the protease granzyme B (GZP) represents an encouraging target for immuno-based imaging; GZP is secreted by cytotoxic CD8+ within the process of immune-induced, caspase-dependent apoptosis. Imaging GZP with ^68^Ga-NOTA-GZP in preclinical models was already able to predict the response to immunotherapy with a high diagnostic accuracy [94].

Moreover, attempts were made to use interferon-γ (IFNγ) immuno-PET (^89^Zr-anti-IFN-γ); first studies showed that ^89^Zr-anti-IFN-γ PET allows imaging of activated lymphocytes in tumoral lesions [95]. Beyond the scope of PET imaging, also promising molecular structures can be targeted using SPECT-ligands; ^99m^Tc-labeled interleukin-2 (^99m^Tc-HYNIC-IL2) allowed the visualization and quantification of tumor infiltrating lymphocytes in the set of patients undergoing immunotherapy; here, ^99m^Tc-HYNIC-IL2 SPECT could be used as a potential imaging approach, e. g., for the differentiation of real as opposed to pseudo-progression in patients undergoing immunotherapy [96].

These promising efforts in both preclinical and clinical setting affirm the further investigation of immuno-PET and the comprehensive translation into clinical imaging to further improve pre-treatment patient selection, response assessment, and clinical management.

### Novel treatments

**Fig. 4:**
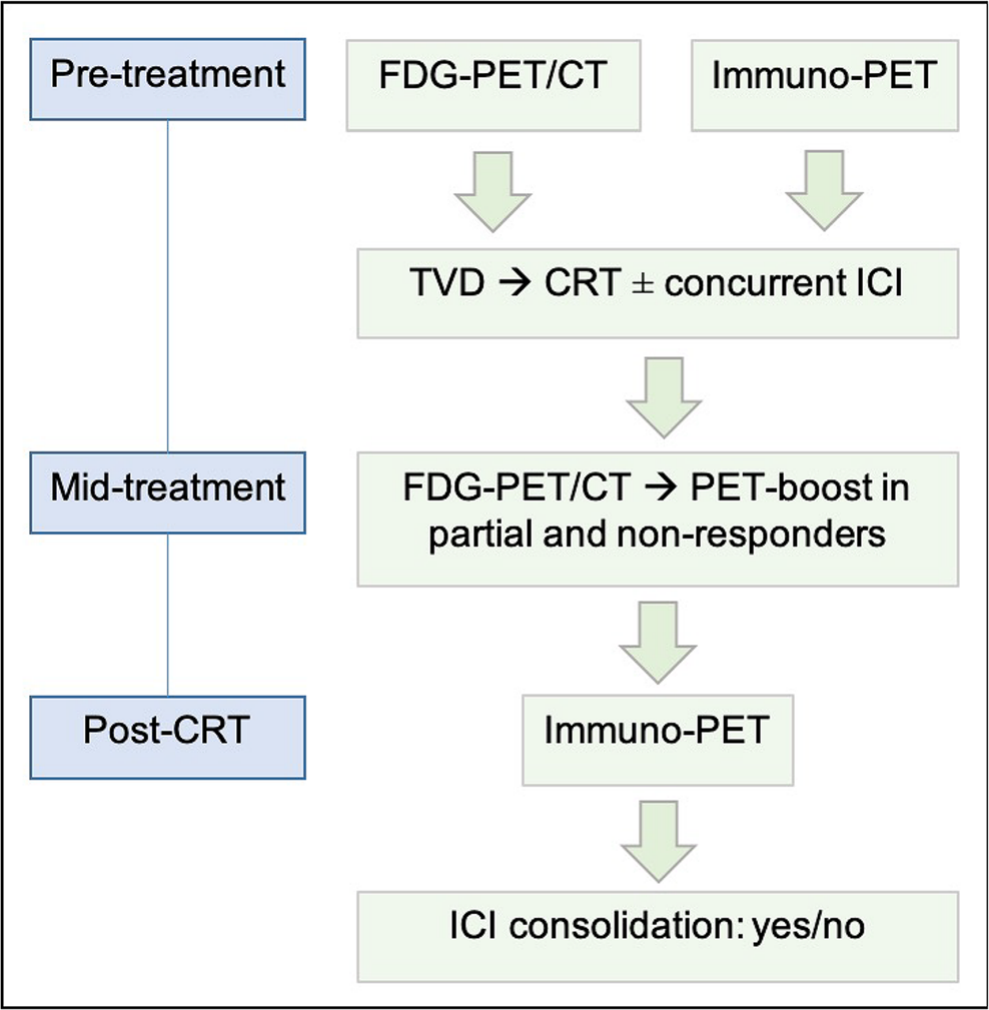
Hypothesis driven imaging paradigm incorporating FDG-PET/CT and immuno-PET in advanced stage NSCLC. Abbreviations: CRT, chemoradiotherapy; ICI, immune-checkpoint inhibitor; TVD, tumor volume delineation

In the coming years, the landscape of immunotherapeutic options for advanced NSCLC will continue to evolve as further drugs gain access to the market. Furthermore, it is yet to be seen if simultaneous ICI with concurrent CRT will further improve patient outcome. It goes without saying that key players have already initiated trials, notably PACIFIC-2 (NCT03519971), KEYNOTE-799 (NCT03631784), and CheckMate73L (NCT04026412) as well as an independent study sponsored by the NCI (NCT04092283) all assessing concurrent platinum-based CRT with simultaneous PD1/PD-L1 inhibition [9]. Strictly hypothesis-driven, potential incorporation of dual PET/CTs in the treatment paradigm for locally advanced NSCLC is a possible strategy predicated on the translation of immuno-PET into clinical practice (Fig. 4).

In metastatic disease, some promising drugs are on the horizon with novel targets. In addition, adoptive cellular immunotherapy is a promising emerging field following the success garnered by some of these agents in hematological malignancies [97]. Taking all these aspects into consideration, there is a need for improved PET assessment, robust criteria, and imaging biomarkers for longitudinal response assessment, characterization of anti-tumor immune response, and acute/late toxicity.

## Conclusion

As novel immunotherapies arise as effective treatment options in patients with stage III/IV NSCLC, new patterns of response/progression and immune-related side effects occur in clinical routine. Response criteria based on morphological features such as RECIST 1.1 have been continuously refined to accommodate these newly occurring, immune-related phenomena. However, hybrid imaging with FDG PET/CT can add comprehensive clinical information beyond the mere morphological changes during immunotherapy and radiotherapy and for the detection of irAEs. As hybrid imaging has shown to significantly influence clinical decision-making in several oncological diseases, it might also allow optimization of immunotherapy and radiotherapy regimens and clinical management in general. Comprehensive inclusion of PET-based response criteria in prospective randomized-controlled trials, but also a general harmonization within the variety of PET-based response criteria is needed to strengthen clinical implementation and wide-spread use of hybrid imaging for response assessment in NSCLC.
